# UNDERSTANDING THE REASONS FOR THE REFUSAL OF CHOLECYSTECTOMY IN PATIENTS WITH CHOLELITHIASIS: HOW TO HELP THEM IN THEIR DECISION?

**DOI:** 10.1590/S0102-67202014000200007

**Published:** 2014

**Authors:** Adilson PERON, Ana Laura SCHLIEMANN, Fernando Antonio de ALMEIDA

**Affiliations:** 1 Faculdade de Ciências Médicas e da Saúde; 2 Faculdade de Psicologia da Pontifícia Universidade Católica de São Paulo - Campus Sorocaba (College of Medical Sciences and Health and 2Psychology College of Pontifical Catholic University of São Paulo - Sorocaba Campus), Sorocaba, SP, Brazil.

**Keywords:** Cholelithiasis, Medical ethics, Informed consent, Behavioral medicine

## Abstract

**Background:**

Cholelithiasis is prevalent surgical disease, with approximately 60,000 admissions per year in the Unified Health System in Brazil. Is often asymptomatic or oligosymptomatic and major complications arise from the migration of calculi to low biliary tract. Despite these complications are severe and life threatening, some patients refuse surgical treatment.

**Aim:**

To understand why individuals with cholelithiasis refuse cholecystectomy before complications inherent to the presence of gallstones in the bile duct and pancreatitis occur.

**Methods:**

To investigate the universe of the justifications for refusing to submit to surgery it was performed individual interviews according to a predetermined script. In these interviews, was evaluate the knowledge of individuals about cholelithiasis and its complications and the reasons for the refusal of surgical treatment. Were interviewed 20 individuals with cholelithiasis who refused or postponed surgical treatment without a plausible reason. To these interviews, was applied the technique of thematic analysis (Minayo, 2006).

**Results:**

The majority of respondents had good knowledge of their disease and its possible complications, were well oriented and had surgical indications by their physicians. The refusal for surgery was justified primarily on negative experiences of themselves or family members with surgery, including anesthesia; fear of pain or losing their autonomy during surgery and postoperative period, preferring to take the risk and wait for complications to then solve them compulsorily.

**Conclusion:**

The reasons for the refusal to surgical resolution of cholelithiasis were diverse, but closely related to personal (or related persons) negative surgical experiences or complex psychological problems that must be adequately addressed by the surgeon and other qualified professionals.

## Abstract (pt)

**Racional:**

A colelitíase é doença de resolução cirúrgica com cerca de 60.000 internações por ano no Sistema Único de Saúde. Muitas vezes é assintomática ou oligossintomática e as principais complicações advêm da migração dos cálculos para as vias biliares baixas. Apesar das complicações serem graves, com risco de morte, alguns pacientes recusam o tratamento cirúrgico.

**Objetivo:**

Entender as razões pelas quais alguns indivíduos com colelitíase recusam a colecistectomia antes que ocorram complicações próprias da doença.

**Métodos:**

Foram realizadas entrevistas individuais segundo um roteiro de perguntas pré-determinadas. Nestas entrevistas procurou-se avaliar o conhecimento dos indivíduos sobre a doença e suas complicações e as razões para a recusa do tratamento cirúrgico. Foram incluídos 20 indivíduos portadores de colelitíase que se recusavam ou adiavam o tratamento cirúrgico sem justificativa plausível. Às entrevistas aplicou-se a técnica da análise temática (Minayo, 2006).

**Resultados:**

A maioria dos entrevistados tem bom conhecimento de sua doença, das possíveis complicações, foram bem orientados e tiveram a indicação cirúrgica pelos seus médicos assistentes. A recusa para a operação foi justificada por experiências negativas próprias ou de familiares com ato cirúrgico, incluindo a anestesia e medo de sentir dor ou perder a autonomia durante o ato cirúrgico e período pós-operatório, preferindo correr o risco e esperar pelas complicações para depois resolvê-las.

**Conclusão:**

As razões para a recusa à resolução cirúrgica da colelitíase são diversas, mas estão intimamente ligadas às experiências cirúrgicas negativas pessoais ou de pessoas relacionadas ou a complexos problemas de natureza psicológica que devem ser adequadamente abordados pelo cirurgião e por outros profissionais habilitados.

## INTRODUCTION

The cholelithiasis occurs in 3-20% of the world population^20^. In the elderly, it is the most common cause of indication for abdominal surgery, with a prevalence of 21.4% in the age group of 60-69 years and 27.5% in individuals over 70 years^14^. It may progress without symptoms for more than half of cases ^13,15^. In 2000, it was estimated that it has affected about 20 million Americans, with direct or indirect cost of six billion dollars^16,19^.

The migration of stone often cause symptoms and can evolve as acute, often severe when not operated. Besides these loco regional inflammatory complications, cholelithiasis may predispose to gallblader neoplasia^17^.

In autopsies of people aged over 75 years, gallstones are even found in 50% of cases^17^. In a Brazilian study, in 4,482 autopsies performed on both men and women, it was found 94 cases of bladder cancer (2.1%), most commonly associated with cholelithiasis cases with an earlier and longer disease diagnose^7^.

The lithogenesis is influenced by multiple factors. The gallbladder stores bile reflux coming from the closure of hepatobiliary bulb and focuses it. The arrival of food in the intestine triggers the neural reflex which adds to the action of cholecystokinin leading to contraction of the bladder, which empties its contents by 75%. When this mechanism does not function correctly, there is stasis of bile, gallbladder distention and ischemia. And it still occurs secretion that activates phospholipase lecithin, promoting inflammation by releasing pro inflammatory mediators^1^. It can be also found bacterial translocation (*E. coli*, *Salmonella sp*) and decrease the amount of liquid, increasing the concentration of bile and favoring the formation of stones^1^.

An extensive literature review, examined the possible non-surgical treatments available for colelithiasis, at that time, such as the use of queno and ursodeoxycholic^2^ acids and extracorporeal lithotripsy by wave shock^4^. None has been found effective in the long term, confirming that cholecystectomy is the only treatment proven effective for solving the colelithiasis^3^.

When cholelithiasis is complicated by cholecystitis, there are no difficulties for the surgery indication by the attending physician, because the picture is very acute, symptomatic and progressive, leading the patient to not discuss the surgical indication. However, in asymptomatic, mildly symptomatic and uncomplicated cases, there are more difficulties in the statement, since some individuals with no pain feel no need of intervention, unless they enter into acute cholecystitis, pancreatitis or colangitis^17^.

In Brazil, the first laparoscopic operation was performed by Thomas Szeco in 1993^5^. Thereafter been reported with increasing frequency by a greater number of surgeons. The introduction of this method allowed it to be taken even in subjects with mildly symptomatic cholelithiasis with less than 1% mortality rate^3^. However, even with all the accumulated knowledge in the field, the precise medical indications and recognition by the patient that the operation is the treatment of choice for the disease, some individuals with gallstones do not accept it.

It's natural to fear and have expectations about the surgery and assume that something may go wrong on the operation, even if it is not an emergency and that will surely enable the planning of the surgery. At this time of conflict, our fears and doubts are coming out much stronger^21^. Thus, it is expected that some individuals refuse or postpone surgical treatment until the moment it is not possible or is beyond your will^18^.

The occasional experience in cases of this nature and reflection on the intriguing decision to patients who refuse operation led to this study, aimed to assess the degree of knowledge that individuals with cholelithiasis who refuse operation have about their disease and its complications and understand the reasons why refuse or postpone surgical treatment and also propose a consent form in language to laity to help them in understanding the disease, reducing their concerns and facilitating therapeutic decision.

## METHODS

The research project and the consent form were approved by the Ethics Committee of the College of Medical Sciences at the Pontificial Catholic University of São Paulo. Participants were not identified at any time of the survey.

It is a qualitative and quantitative research, based on interviews with 20 patients suffering from cholelithiasis, despite medical indication for performing cholecystectomy, did not accept it even with the guidance of professional or chose to postpone it. During the review process, some categories of reasons were identified to postpone or reject the operation. These categories are shown in Figure 1 ^21^.

**FIGURE 1:** Categories of the justifications for refusing or postponing elective cholecystectomy

a) Psychological: relate the emotional experiences, complications and they are subjected
b) Educational: define the forms of formal and informal learning
c) Social: relationships which people live and express with their counterparts in the various scenarios in which transitions, such as family, school, work and interpersonal relationships
d) Cultural: to understand the world through their experience with their community of origin or current
e) Religious beliefs: relation with the sacred and the notion of transcendence and finitude
d) Fear: disruption at the idea that one is exposed to some kind of real danger or not
e) Anxiety: uneasy feeling of discomfort or fear with autonomic response (the source is not specific or unknown to the individual)
f) Fear and anxiety about the unexpected operation: invasive process that generates pain, physical suffering and so the emotional aspects are mobilized
g) Fear and anxiety about the family history: negative experiences with the medical environment , or when final action was considered negative
h) Fear and anxiety in the face of suffering: generated by the possibility of suffering with anxiety, which can worse on the experience of real suffering
i) Fear and anxiety before death: the notion of subjectivity and finitude hurts belief that the human being is et ernal

These categories do not exhaust the possibilities of understanding about the difficulties of individuals to make a decision about the surgical procedure without being under pressure and time urgency, but these were chosen by the researchers, having considered their emotional impact.

The survey was conducted with 20 participants of both sexes, aged 19-70 years and diagnosed with cholelithiasis, confirmed by abdominal ultrasound or other diagnostic imaging method , with the classic symptoms of the disease with few symptoms or " asymptomatic " . Inclusion criteria for the study were: 1) presence of calculus in the gallbladder diagnosed by imaging method) ; 2) denial of the individual to perform elective cholecystectomy or successive delay of surgery for poorly justifiable reasons; and 3) have agreed to participate in the study by signing the consent form. The number of participants was defined by "saturation criteria" proposed by Muchielli^12^.

Participants in this study were recruited from the private clinic (n=11) and Outpatient Specialty Health Network from the city of Sorocaba (n=9). After identifying the patient, the nature of the study was explained to them and then was applied the term of consent and their signatures, harvested according to the Resolution 196/96. The interviews were conducted in office.

To investigate and understand the reasons for the refusal to accept surgical treatment, interviews with all participants made ​​individually through open-ended questions, were conducted according to a pre established route using a tape recorder to record the narratives. The script was: 1) What were your symptoms at diagnosis time and current symptoms?; 2) Which were the methods for the diagnosis of cholelithiasis?; 3) Who indicated surgical treatment?; 4) What do you know of this disease?; 5) What do you expect to happen if you operate?; 6) What are your reasons for not performing the surgery?

In the qualitative analysis of the interviews, was sought to privilege the speech, concepts and thoughts in order to establish more detailed and in-depth exchanges on the topic in question, its causes and consequences of the negative decision to perform the operation.

Quantitative analysis was performed descriptively aiming to inform the frequency with which the ideas appear in the speeches and representations. Developments within the follow-up period were also analyzed descriptively and quantitatively.

The interviews were transcripted and initially analyzed individually. From the analysis of speech according to Badin and Minayo^11^ the ideas presented by the participants were identified that represented relevant points in speeches, influencing their decision facing the medical indication for surgery. After individual analysis, other was also made with all the information and representations provided by the participants, identifying common or correlated factors in these discourses.

### Adaptation of educational material

Having as starting point the informed consent already provided by the Brazilian College of Digestive Surgery - CBCD was chosen to adapt it to the cultural reality of the participants of this research and including information that was considered to be important to help patients in therapeutic decision. It is noteworthy that for this purpose the consent of CBCD for use and adaptation of the instrument was obtained.

## RESULTS

The results are presented in tables and divided according to their content. Table 1 presents the characteristics of the study participants. It may be noted that the percentage of women (80%) exceeded that of men (20%) and the predominant age group is between 36-60 years (80%). The time of diagnosis was more prevalent between three months to one year (75%). Most participants had mild symptoms (n=18, 90%) and 30% had some metabolic change.

**TABLE 1:** Characteristics of participants

Parameter	Categories	Number
Ages	19 to 35 years 36 to 60 years Over 60 years	2 (10%) 16 (80%) 2 (10%)
Gender	Feminin Masculin	16 (80%) 4 (20%)
Ethnicity	White	20 (100%)
Religion	Catholics Non catholics	11 (55%) 9 (45%)
Marital Status	Married Widowed Did not answer	8 (40%) 2 (10%) 10 (50%)
Diagnose time	From 3 to 6 months From 6 months to 1 year More than 1 year	9 (45%) 6 (30%) 5 (25%)
Symptoms	Mild* Asymptomatic	18 (90%) 2 (10%)
Associated diseases and status	Gastritis Hypertension Irritable bowel syndrome Diabetes mellitus Dyslipidemia Hypothyroidism Pregnancy Intestinal neoplasia Irregular heartbeat	6 (30%) 5 (25%) 5 (25%) 3 (15%) 3 (15%) 1 (5%) 1 (5%) 1 (5%) 2 (10%)
IMC	Between 28 to 35	Average of 32,5 Kg/m2

* Mild symptoms=sporadic biliary colic, dyspepsia or pain in the upper abdomen without the need for urgent care

To better understand this refusal of surgical treatment, was used the technique of individual interviews, providing a free and appropriate environment for the participant to have full opportunity of expression, also avoiding interfering with its reflection.

Table 2 presents a summary of the responses to the question "what were your symptoms at diagnosis and current symptoms?"

**TABLE 2:** Most common symptoms presented by participants

Symptoms	Number
Vomiting and nausea	15 (75%)
Dizziness and fainting	14 (70%)
Strong abdominal pain	12 (60%)
Stomach pains, reflux and bloating	12 (60%)

The related complaints of abdominal distension, nausea, vomiting, dyspepsia, food intolerance, intestinal pain in the epigastrium, composed the universe of symptoms that often led the individual to seek specialists in Gastroenterology; they report vague complaints that often did not permit correct diagnosis of cholelithiasis or with exams inducing only to functional disorders, "gastritis" or "colitis " with ultrasonography always normal.

The next question was: "Which were the methods for the diagnosis of cholelithiasis?" Ultrasonography was the most used method (90 % of cases) followed by tomography of the abdomen in 10%, indicated on the possibility of pancreatitis and biliary tract dilatation.

"Who recommended surgical treatment for gallstones?" Medical emergency service indicated it in 25% of cases, and the other 75% by medical assistants. The correct indication of the treatment was taken in all cases, but the patients sought other professionals hoping different diagnosis and treatment.

The following question "What do you know about your illness?" showed that 55% express great knowledge to the point of describing it in details, including complications such as pancreatitis, choledochal calculus, bladder perforation and other complications, but still did not want to be operated. The other 45% had little or no knowledge at all about their disease.

Referring to the question "What do you expect to happen if you operate?" most replied that they hoped to get the urgency status (90%). They expected a decision that was "alienated form of Medicine" or "by imposition of fate", resulting in their own complications of the disease that "force" to be treated surgically. Even though the higher risk, preferred them to an imposed decision.

"What are your reasons for not performing the surgery?" The majority (65%) reported being afraid of anesthesia or complications and 35% justified their decision by a history of traumatic experiences, whether personal or with people of their coexistence. Some have also had contact with death or suffering of a loved person, showing not only fear, but also anger and little hope. The data analysis also revealed that there are some justified concerns that should be clarified in a written document, aiding them in the treatment decision. Among these are: 1) return to work without restrictions; 2) return to sexual activity; 3) feeding; 4) commonly associated diseases, such as diabetes mellitus, hypertension, hypothyroidism requiring continuous medication, are also frequent concern.

### Statement of Informed Consent

The CBCD document brings enlightening information about the surgical procedure; but was noted that many of the issues raised in the interviews of this study were still open. Thus, adaptation from the original consent form that by request of the CBCD could not be copied in its entirety, resulted the model of Figure 2, as a proposition to be used by patients with cholelithiasis.

**FIGURE 2:** Statement of informed consent adapted for this study from the one published by Brazilian College of Digestive Surgery - CBCD

What is the gallbladder?
It is a kind of bag that receives and concentrates the bile fluid produced in the liver, functioning as a reservoir for bile. It has the function of helping the digestion of some food, especially fat. Thus, bile is released mainly when we eat these foods and launched in bile duct (common bile duct) which is the tube that carries bile from the gallbladder and liver to the intestine, opening into the intestine along with the pancreatic duct that carries the secretion that comes from the pancreas and helps the digestion of protein foods.
As the stone (calculus) is formed in the gallbladder?
Bile contains several substances, including salts of cholesterol and pigments; so, it has a dark green color. When some of these substances increase in quantity in bile, they can deposit and over the months and years; these deposits come together and form stones (calculi). Other parts of the body such as the kidneys, the bladder and the channels of saliva, tear can also form stones (calculi). But the stones of these locations are different from the gallblader.
How are the stones (calculi)?
The number, size, shape and color of gallstones are quite variable. Some people only have one stone, while others have several . They can also vary in size from tiny, as a grain of sand, to large as an orange.
Who can have stone (calculus) in the gallbladder?
Stone or calculus of the gallbladder is a very common disease. About 10% of people have gallstones. Over 10 million Brazilians have this problem. Anyone can have gallstones, but some are more likely, according to certain variables such as: 1 ) age (the disease can affect even children, but its frequency increases with age and is more common in adults and elderly); 2 ) woman (the gallstones are more common in women than in men, especially in already became pregnant); 3) obesity (fatter, greater is the possibility of having gallstones; however, lean people can also have calculi); 4 ) heredity (the people who have family members with calculi have more chances of having this disease than those without).
Symptoms and complications caused by gallstones
Many individuals who have gallstones have no symptoms. Whether a person has no symptoms or complications depends on the number or size of the stones. Sometimes a single small stone can cause serious complications, as, for example, acute pancreatitis. The presence of gallstones may cause mild, serious or intense symptoms, the most common being : 1) severe pain in the abdomen (belly), usually on the right side or in the region in the stomach; it lasts for minutes to hours, but when longer may indicate complication; in this case, you should seek medical attention urgently; 2) nausea (feeling sick) and vomiting, especially when eating certain foods (fat, eggs, banana, etc.); 3) inflammation or infection of the gallbladder (cholecystitis), in this case, besides the pain other symptoms may appear such as fever, nausea and prostration and severe condition that requires emergency care, because the bladder may even rupture putting your live at risk; 4) jaundice (yellowing of the white parts of the eyes and skin) usually occurs when the tube that carries bile to the intestine (common bile duct) is blocked by stone, then the bile is accumulated in the liver, the blood flows back and makes the skin yellow; 4) acute pancreatitis (inflammation of the pancreas), which is caused by stone that jammed where flow into the biliary and pancreatic channels, causing blockage of both leading to inflammation and infection of the pancreas, which is also a major complication of the disease.
Diagnosis
The best method for diagnosing gallstones is an ultrasound of the abdomen. The scan may not show the stones in many patients.
Treatment
The only form of effective treatment for gallstone or calculus is the surgical removal of the gallbladder (cholecystectomy). Other treatments like lithotripsy (“ break the stone “ with special equipment) and drugs to dissolve the stones, have been tested, but did not give good results and should not be used because only delay proper treatment. Same is said to magic recipes, teas, feeding recommendation done by lay people based on personal experiences, popular beliefs and feeding restriction as they will only delay definitive diagnosis and treatment, which is surgical removal of the gallbladder. The removal of the gallbladder through laparoscopic surgery is possible in most patients (“little holes surgery”). Initially, gas is injected (carbon dioxide) into the abdomen (belly) to create a space where the surgeon can perform the operation safely. After conducting four few millimeters holes, a television camera is placed inside the abdomen through one of the holes so that the surgeon and his team can view the entire abdomen on a TV. The tools to perform the operation (forceps, scissors, suture materials, etc.) are placed through the other holes
All people who have gallstones need to operate?
People who already have symptoms should be operated, due to frequency of complications that in these situations is very high. The people who have yet life expectancy of many years have higher risk of complications, even without symptoms. The elderly and other associated diseases (diabetes, high blood pressure, heart complications) the surgeon and the clinical need to assess the risks and discuss with the patient to make the decision about the operation. Even people who have other diseases, and if they are well controlled, the risk of complications is small. However, under conditions of acute inflammation and infection of the gallbladder (cholecystitis) operation becomes imperative in emergency.
After removal of the gall bladder, will I have any restrictions in my diet?
You do not need to modify your diet after the operation, because the gallbladder have little important function in the body, which is to store bile. It do not produces bile, only helps in storage. The production of bile by the liver remains normal after removal of the gallbladder. No sequel or consequence to the organism exists after removal of the gallbladder. Thus, most people who had intolerance to certain foods, nausea , vomiting, discomfort or pain after eating, with the removal of the gallbladder these symptoms disappear or greatly improve. However, there is a group of people (approximately 20%) in whom symptoms persist after the operation, but the risk of having the aforementioned complications is reduced.
Surgical treatment
**Postoperative recovery:** Most patients stay in the hospital only one day and can return to work and to all activities, including sports, in a week or two. **Complete and final resolution of the disease:** In some people the symptoms remain. **Little pain in the postoperative period:** As laparoscopic operation is minimally invasive and almost doesn’t damage the tissue around the bladder, pain in the postoperative period is discrete and improves with common analgesics. **Minimal surgical scar:** Are performed only four holes whose scars may disappear after sometime **Risk of infection and complications:** When the operation is done on a scheduled basis (elective), the risk of infection and other complications is small, around 0.1%. However, when performed in emergency and due to complications (cholecystitis) , the risk is higher (up to 3%) and hospitalization time and recovery will also be longer. **Risk of death:** It is very low. Even in elderly individuals the risk of death related to the transaction is less than 0.5%. However, if the operation is done in emergency, infection or other complications, the risk of death rises to 1%.
Is the operation 100% safe?
Although the results of surgical treatment are excellent, some patients may have complications, as with any surgical procedure. The most common are: viscera injury, infection, bleeding and anesthetic risk (low). If you cannot perform the operation by laparoscopic technique (“holes technique”), you may need to make a larger incision (cut) in your abdomen to complete the operation. The risks of the operation are more common in patients who have severe disease or complications such as inflammation of the gallbladder, jaundice, acute pancreatitis at the time of operation. In these situations, the operation is generally more difficult to achieve and should be done as emergency.
Comorbidities (accompanying diseases)
As the disease affects more frequently the older people and some of them favor the formation of gallstones, there are common associated diseases. Among them the most common are obesity, diabetes and dyslipidemia (high cholesterol) . But also high blood pressure, heart problems and osteoarthrosis are common diseases in the elderly. All these associated diseases should be adequately controlled before surgery. In these cases, the set of a clinical medical care with the surgeon and the anesthesiologist will ensure good control of associated diseases before operation, ensuring maximum patient safety.
Anesthesia and pre-anesthetic evaluation
Many people are afraid or apprehensive and anxious before the anesthesia. Most of the time it is generated by ignorance of the subject, bad reports of relatives, friends or even by the press and broadcast media. Long time anesthesia is procedure completely safe for the patient; the medications and anesthetics had advances. There is no reason to think that you will be subjected to anesthesia and “will not wake-up “ as some argue. Your whole body is monitored during the anesthetic doctors team, nurses and technicians. Pressure, breath and heart rate are carefully monitored by the team and devices with automatic alarms. Furthermore, some believe which during anesthesia dreams, nightmares and unpleasant memories occur is fantasy. None of this happens. You sleep relaxed and wake-up as if nothing had happened, rested. An appropriate pre -anesthetic assessment will bring peace and security to you, to surgical and anesthesia teams. In this pre-anesthetic consultation, you can talk with the anesthesiologist, explain your fears and concerns and he will know guide you, answer your question and everyone will be safer during the procedure. Many studies have identified the major fears or concerns related to anesthesia, they are: fear of not sleeping; feeling pain during the operation; feeling cold and shivering; feel something and not be able to communicate; surprising the environment of the operating room; fear that anesthesia finishs before the end of the operation; fear of having “allergy” and “anesthetic shock”; fear of not waking-up after anesthesia and fear of postoperative pain. Of all these, the only real risk is having to allergic reaction to the medication, but that is very rare and can happen to any medication you use. The difference is that by being in the presence of a medical team and all their vital signs monitored, you will have chance to be attended to immediately and with less risk. Cholecystectomy, the operation you need to do, requires general anesthesia. Before entering the operating room, you may have already received slee -inducing drug that has fast action. General anesthesia prevents you from feeling pain. After the operation you receive another medicine that makes you wake-up and everything goes like you slept peacefully in that period. Don’t doubted it: during the entire period of the operation you are being carefully controlled by the anesthesiologist.
Postoperative guidance
The recovery is usually very fast and most people return to their normal activities within a few days. The guidelines should be followed so that you have little discomfort and recovery occurs uneventfully. It has no special diet. You can eat or drink any food you want, including solid foods. Some patients may possibly have nausea and vomiting on the first day after surgery due to drugs and anesthetics received. If you have nausea and vomiting, ingest only liquids in small quantities at a time. These symptoms usually disappear within one or two days after the organism to eliminate drugs received in a hospital. If nausea and vomiting persist after this period, see your doctor. Shoulder pain is common after this type of operation. It is caused by the gas injected into the abdomen and it irritates a nerve diaphragm which lies between the abdomen and thorax. It is not due to twist or sprain in the shoulder. Shoulder pain usually disappears in a few hours or days. If it is severe, take analgesic (pain medication) prescribed by your doctor. The cuts (holes) will be closed with stitches and covered with simple dressing (micropore). It is common to have bruises (“blue” or “purple”) or minor bleeding. This is normal and you should not worry. Do not remove the micropore unless your doctor the tells you to do. You can take a full bath and wet the micropore. Dry the belly with a towel. Usually, the patient carries on without the need for special care on the cuts. However, if it has the appearance of infection (red with pain, discharge of pus or strong-smelling), contact your doctor. Breathe deeply three times every hour to best expand your lungs and avoid complications, such as fever and pneumonia. Avoid staying long lying down or sitting. Try to walk several times a day. If you can, walk, climb stairs or even run. There is no danger on doing that. Once you’re moving fast and with little pain, you are able to drive. You can lift up to 20 kg in the first month and after this time, you have no more limitations.

### Clinical evolution of the study participants

Although all participants, in principle, refused to perform the operation, more than half were operated, most due to acute complications of the disease (n=8, 40%) and others have decided to operate electively (n= 5,25%).

## DISCUSSION

Despite the term of informed consent being the most informative and complete as possible, it is not definitive, nor relieve the doctor of effective participation, clarifying doubts of the patients, reviewing and discussing points in which patients have difficulty, allowing time for the patient to think, remarking further consultations to resume the subject, effective participating in the process and not just signing it. In this way, the doctor-patient relationship is of great importance because it strengthens the relationship and brings us closer to our patients, leaving them less anxious about the risks of the operation and assisting them with the new situation before the surgical procedure.

This study used a sample of patients that resembles the general population of patients with cholelithiasis^3,9,10,17^. The model is a qualitative study, based on analysis of interviews with pre-established script allowing to explore the causes that have led some patients to refuse or postpone the operation without justification. The data showed that most participants had good knowledge of the disease and its complications. They also reported they were well informed by their physicians in the diagnosis and follow-up process, but chosen to delay surgery, justifying this decision mainly by fear of anesthesia, the surgery and the postoperative period. However, other concerns that have contributed to this behavior, including early concern in pain, and having other symptoms that may affect their lifestyles, leaving often the procedure for an urgent emergency situation, which may impair resolution of their disease.

During the analysis of this study it was felt that patients will undergo a surgical procedure in general and in particular those who have difficulty deciding for surgical treatment, need to be fully educated, especially in what surround the procedure, the information of the causes of the disease, its treatment options, risks and benefits of the proposed treatment and changes in the individual's routine pre- and postoperatively.

Though fear - main reason for refusing to surgical treatment - is not rational sense, it is understood that patients like detailed clarification material with technical explanations in language addressed to the lay, can be part of a process of clarification and demystification of everything surrounding the procedure, helping them to accept the operation. This study presents a proposal for informed consent with all the information about the operation, its risks, pre-and post-operation, as well as shown in friendly format helping the patient in his decision. Recently, associations and specialist services began to use the Statement of Informed Consent that instructs and informs patients and families about the procedure to be performed, but its primary purpose is to safeguard the professionals involved in the procedure for any future demands inherent complications the procedure and even malpractice.

Despite the informed consent being the most informative and complete as possible, it is not definitive, nor a relief for the doctor of effective participation, clarifying any questions, reviewing and discussing points in which patients have difficulty, giving time to think, redrawing new queries to resume the subject. In short, effectively participating in the process and not just signing it. Kubler-Ross^8^ introduced the concept that when dealing with stressful situations, such as loss, grief, tragedy or incurable diseases, go through different stages to reach acceptance, more advanced stage. This researcher has defined five common stages of this process that are: "denial," "anger," "bargaining", "depression" and "acceptance"^8^. The author maintains that all persons subject to these situations have at least two of these stages, which do not always occur in this order, and may also be advance or throwback. In this sense, the doctor-patient relationship is of great importance, because it strengthens and approaches patients, leaving them less anxious about the risks of the operation and assisting them with the new situation before it comes. On experience with this small group of patients, who earlier did not accept surgical treatment, a part of them (25%) finished undergoing elective operation, achieved with the help of the doctor the right decision.

Evidently such an instrument should not be the only form of aid to the patient. In this sense, establish a good relationship of doctor-patient trust and assistance of trained professionals working these past traumas are key to providing security to the patient at this time.

## CONCLUSIONS 

All patients who refused or delayed the operation expressed many fears - including death - and loosing self-control. These feelings were not due to lack of information. It was determined that other personal factors contributed to increase insecurity, which contributed to the decision of making or not the operation. The proposal is to use this or other informed consent, incorporating all the information that may help in the decision. Moreover, good and lasting doctor-patient relationship and the availability of more time for decision were important factors for a good part of the participants that accepted to perform the operation electively, after reading and understanding the informed statement.
